# Dimensional Accuracy and Mechanical Properties of Chopped Carbon Reinforced Polymers Produced by Material Extrusion Additive Manufacturing

**DOI:** 10.3390/ma12233885

**Published:** 2019-11-25

**Authors:** Evren Yasa, Kıvılcım Ersoy

**Affiliations:** 1 Department of Mechanical Engineering, Faculty of Engineering and Architecture, Eskişehir Osmangazi University, Eskisehir 26180, Turkey; 2 FNSS Defence Systems, Ankara 06830, Turkey; kivilcim.ersoy@fnss.com.tr

**Keywords:** fused filament fabrication, tensile testing, anisotropy, chopped carbon reinforced composites, dimensional accuracy

## Abstract

Fused Filament Fabrication (FFF), classified under material extrusion additive manufacturing technologies, is a widely used method for fabricating thermoplastic parts with high geometrical complexity. To improve the mechanical properties of pure thermoplastic materials, the polymeric matrix may be reinforced by different materials such as carbon fibers. FFF is an advantageous process for producing polymer matrix composites because of its low cost of investment, high speed and simplicity as well as the possibility to use multiple nozzles with different materials. In this study, the aim was to investigate the dimensional accuracy and mechanical properties of chopped carbon-fiber-reinforced tough nylon produced by the FFF process. The dimensional accuracy and manufacturability limits of the process are evaluated using benchmark geometries as well as process-inherent effects like stair-stepping effect. The hardness and tensile properties of produced specimens in comparison to tough nylon without any reinforcement, as well as continuous carbon-reinforced specimens, were presented by taking different build directions and various infill ratios. The fracture surfaces of tensile specimens were observed using a Scanning Electron Microscope (SEM). The test results showed that there was a severe level of anisotropy in the mechanical properties, especially the modulus of elasticity, due to the insufficient fusion between deposited layers in the build direction. Moreover, continuous carbon-reinforced specimens exhibited very high levels of tensile strength and modulus of elasticity whereas the highest elongation was achieved by tough nylon without reinforcement. The failure mechanisms were found to be inter-layer porosity between successive tracks, as well as fiber pull out.

## 1. Introduction

For many industries where lightweight applications are becoming more important, such as aerospace, automotive, marine, nuclear, and biomedical industries, combining the advantages of Additive Manufacturing and composites has the high potential to provide strong opportunities. The worldwide demand for lightweight PMCs (polymer matrix composites) is growing. For example, almost 50% of aircraft frames are produced from composite materials, whereas, in the automotive industry, the annual growth in composite materials exhibits a 5% increase due to their good mechanical properties, flexibility in design and high performance [1]. However, efficient fabrication methods for composites still possess some problems. Additive Manufacturing (AM) is defined as the “process of joining materials to make parts from three-dimensional (3D) model data, usually layer upon layer, as opposed to subtractive and formative manufacturing technologies” [2]. AM has many advantages over conventional processes such as reduced lead time from design to testing, high level of customization and automation, and the ability to fabricate very complex designs which may not be possible otherwise. While AM is a cutting-edge technology with a wide range of applications, there are several barriers hindering its growth, such as cost of equipment and materials, imperfections like voids, stair-stepping effect, long production times, and material and size limitations [3]. The most widely preferred AM process for PMCs is the Fused Filament Fabrication (FFF) method. In FFF, a feedstock filament of the raw material on a spool is fed into the extrusion head and a heating chamber liquefies the solid material before it is selectively deposited. 

One of the most comprehensive studies in this field carried out by Tekinalp et al. focused on chopped carbon-fiber-reinforced acrylonitrile butadiene styrene (ABS) polymers at different fiber loadings in order to evaluate the potential for load-bearing components. The tensile testing results showed that FFF composites have a significant porosity problem as compared to compression-molding specimens, whereas a high fiber orientation in the printing direction was encountered with FFF [4]. The tensile properties of ABS reinforced with glass fibers were studied by Zhong et al., showing that the glass reinforcement could improve the tensile strength and surface rigidity at the expense of flexibility and handleability [5]. Ning et al. [6] fabricated carbon-fiber-reinforced ABS specimens by varying the carbon-fiber content between 0% and 15%. The carbon reinforcement increased the tensile strength and Young’s modulus, whereas the toughness, yield strength and ductility were reduced. Additionally, porosity was encountered as a severe problem. There were some other studies focusing on evaluating whether a polymer feedstock is a good candidate for material extrusion methods. Duty et al. developed a practical model taking typical printing parameters into account to check conditions for printability such as pressure-driven extrusion flow, bead formation, bead functionality, clogging, etc. [7]. One of the very widely used matrix materials used with FFF is Poly Lactic Acid (PLA). Ferreira et al. presented a mechanical characterization and SEM (Scanning Electron Microscope) micrography of PLA reinforced with short carbon fibers [8]. The results showed that the tensile modulus and shear modulus of reinforced PLA was increased in comparison to pure PLA. Moreover, consistently with other studies in the literature, failure in reinforced PLA happened at lower strains than in pure PLA leading to the fact that the reinforced material became more brittle with the addition of short carbon fibers. Different reinforcement materials were also studied with PLA matrices. Liu et al. studied the effect of wood, ceramic, metal and carbon fiber reinforcements on the mechanical properties with different raster angles. Regarding formability, it was shown that wood-based PLA was the most difficult due to a delamination effect. However, in this study, it was found that lower mechanical properties were obtained with carbon and wood reinforced PLA due to high porosity, poor compaction, and poor adhesion between filaments compared to pure PLA [9]. The fracture toughness in relation to the fiber content in PLA specimens was studied by Papon and Hague [10]. It was shown that thermoplastic polymer (PLA) reinforced with short carbon fiber had increased fracture properties (fracture toughness and energy release rate) in comparison to the baseline polymer when the fiber content was set to 5%. The most critical factors for the fracture toughness seem to be the bead layup sequence, fiber pullout, interfacial de-bonding, and void formation. Higher fiber contents did not significantly alter the fracture toughness due to higher intra-bead voids, microcracks, and poor interfacial bonding. There were also some studies investigating the effect of FFF process parameters. For instance, Rao et al. investigated the effect of layer thickness, print temperature and infill pattern on the tensile strength of carbon-fiber-reinforced PLA [11]. The results indicated that the interactions between layer thickness and infill pattern, and between infill pattern and extrusion temperature had significant effects on tensile strength. The lowest layer thickness, being the most influential factor as expected, led to the highest tensile strength due to higher bonding area between layers. In a recent study by Yasa, it was shown that build orientation has a significant influence of carbon-reinforced tough nylon. The impact toughness of specimens built vertically was reduced by 90% in comparison to other directions where the impact was not received in between deposited layers [12].

More recently, studies on embedding continuous fiber in plastic materials were realized mainly using FFF for different applications [13,14,15,16,17,18,19,20,21]. Various matrix materials such as Polyamide (PA), nylon, ULTEM®, PLA, and Polypropylene (PP) are used whereas the most commonly used reinforcement material is carbon. The studies mainly focusing on tensile and flexural properties of continuous fiber-reinforced polymers showed that there are some limitations, such as adhesion between fibers and the matrix, weak bonding, porosity, problems due to irregularities and discontinuity of the fibers as well as cutting, etc. However, it was seen that the strength increases significantly while toughness decreases as a result of continuous fiber reinforcement. In order to overcome some limitations, Rarani et al. assessed the quality of fused deposition modeling of continuous carbon-fiber-reinforced PLA with a new extruder design. The experimental results indicated that the tensile and bending strengths were increased by up to 35% and 108% in comparison to pure PLA, while the predominant failure modes were delamination and delamination-induced-matrix cracking [22]. Tian et al. showed that temperature and pressure were critical parameters for the forming process determining the mechanical properties when 3D printing with continuous carbon fiber-reinforced PLA [16]. In another study by Tian et al. [23], three-dimensional (3D) printing of recycled carbon fibers was studied. An impregnated carbon fiber filament was obtained after recycling 3D printed carbon-fiber-reinforced thermoplastic composites without sacrificing the fiber properties. Despite the fact that aging of the matrix was encountered, comparable and even higher mechanical properties, such as 25% improvement of flexural strength, were achieved using the remanufactured composite specimens in comparison to the originally 3D printed composites. The study by Mori et al. on the tensile and fatigue testing of carbon-fiber-reinforced ABS material showed that thermal bonding was critical for the increase in mechanical properties [24].

This study mainly focuses on investigating the mechanical properties of tough nylon produced by the FFF method with the reinforcement of chopped carbon fibers, taking different build directions and infill ratios into account, in comparison to no reinforcement and continuous carbon-reinforced specimens. Moreover, dimensional accuracy and geometrical features of built benchmark specimens were investigated.

## 2. Materials and Methods 

**Figure 1:**
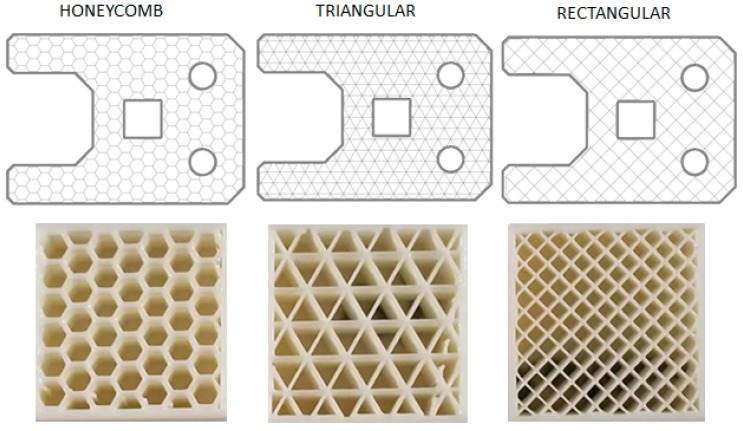
Various infill strategies [26,27].

**Figure 2:**
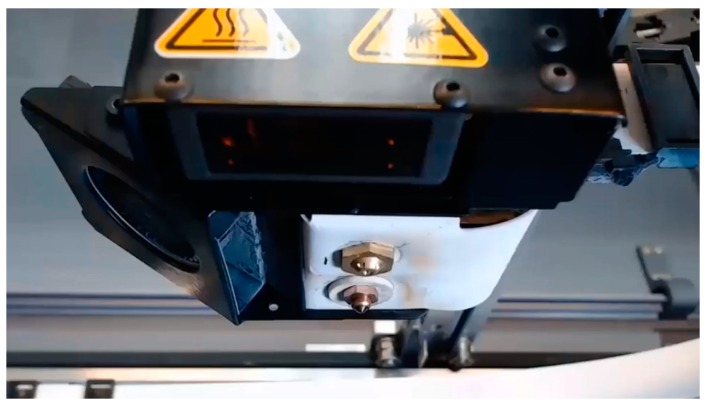
Plastic and fiber nozzles on Mark Two® equipment [29].

All the specimens used within this study were produced on a Markforged Mark Two® equipment with standard parameters from carbon fiber-reinforced tough nylon, commercially known as MarkForged Onyx. The maximum size of the print volume of this printer was 320 × 132 × 154 mm. The Onyx material is tough nylon pre-impregnated with chopped microcarbon fibers in the filament form, combining the toughness of nylon with the thermal properties of carbon [25,26]. Various infill strategies can be used with FFF as depicted in Figure 1 [26,27]. For 100% dense parts, a rectangular infill is generally preferred, and the deposition orientation is varied from layer to layer, whereas for lower densities, honeycomb or triangular infills can be used for weight reduction. This is probably due to the fact that a rectangular pattern allows an infill density of 100% because it does not self-intersect inside the layer [28]. The nozzles used in Mark Two® are shown in Figure 2. One of the nozzles is used to print plastic or Onyx fiber whereas the other is used for continuous fiber replacement [29]. The fiber nozzle is different from usual filament extrusion heads due to its cutting mechanism for cutting the fiber.

**Figure 3:**
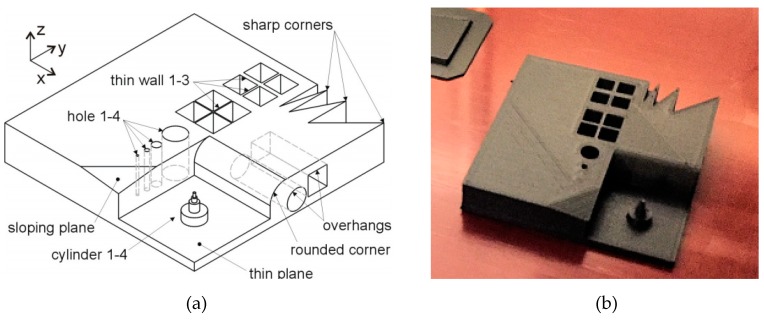
Benchmark-1 geometry [30] (**a**) Built specimen on the print bed (**b**).

**Figure 4:**
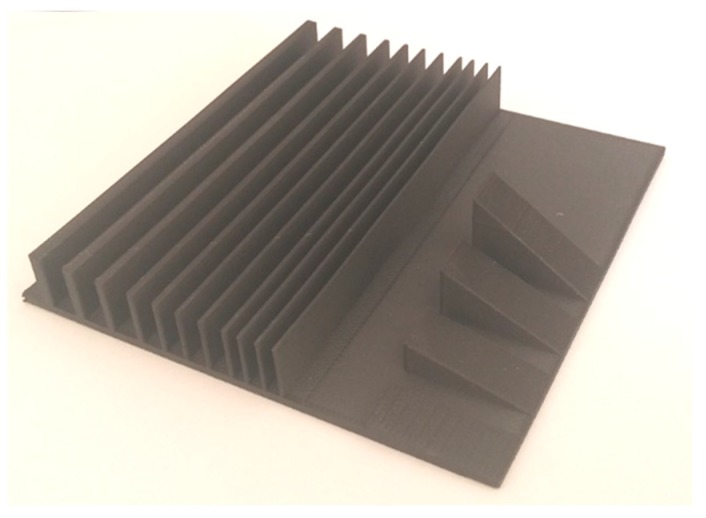
Benchmark-2 geometry used to test the capability to produce thin walls.

Firstly, the benchmark specimens were built to test the capability of dimensional accuracy and producing proper geometrical features. In order to understand the minimum wall thickness achievable with FFF of Onyx material, as well as other geometrical limitations, a benchmark geometry, which is well known in the AM of metals, was used as shown in Figure 3a [30]. There were many features on this benchmark ranging from sharp corners to thin bosses, holes, inclined surfaces, etc. It was manufactured with a 50% triangular infill strategy for maximum dimensional/geometrical accuracy as shown in Figure 3b. On the second benchmark (see Figure 4), walls with thicknesses of 0.3–3.0 mm were produced with a height and width of 12 mm and 100 mm, respectively. Moreover, the stair effect was studied on inclined walls with different inclination angles (5–35 degrees).

**Figure 5:**
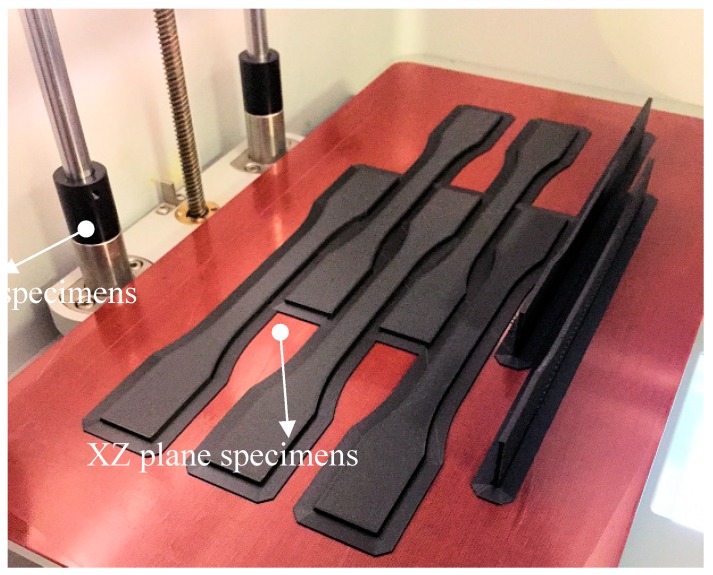
The flat (XY plane) or standing (XZ plane) specimens.

Tensile specimens were manufactured from Onyx and tough nylon material in addition to continuous carbon-fiber-reinforced nylon. A total of 30 specimens were produced under six different configurations as shown in Table 1. The first specimens (E_Nylon_R_100_XY) were built as lying specimens from tough nylon only without any reinforcement at 100% density. Moreover, specimens were built in two directions, either lying (XY plane) (A_Onyx_R_100_XY) or standing on their long side (XZ plane) (B_Onyx_R_100_XZ). The other build direction could not be tested due to the maximum build volume of the equipment. The fabricated specimens on the print bed are shown in Figure 5. The use of a brim, the surrounding peripheral deposition around the specimens, was necessary as an anchor of print bed, which is especially critical for parts tending to warp. Brims were used in producing tensile specimens to increase the area of the first layers as a precaution to deformation. In addition to the build direction, the density effect was also tested at 75% (C_Onyx_T_75_XY) and 50% (D_Onyx_T_50_XY) infill density values with lying specimens. Lastly, lying tough nylon specimens (F_Nylon_CF_R_100_XY) were produced by concentric reinforcement of continuous carbon fiber.

During tensile testing, the EN ISO 527-4 standard entitled “Determination of tensile properties of plastics Part 4: Test conditions for isotropic and orthotropic fiber-reinforced plastic composites” was used. The tensile test equipment was a universal Zwick-Roell equipment with a loading capacity of 250 kN. After the tensile testing was complete, the broken specimens were investigated by optical microscopy and scanning electron microscopy. For the hardness testing, a hardness tester from Bareiss Digi Test was used to measure Shore D hardness as per the TS EN ISO 868 standard with a contact pressure force of 5100 g. 

## 3. Results

### 3.1. Dimensional Accuracy and Geometrical Features

**Figure 6:**
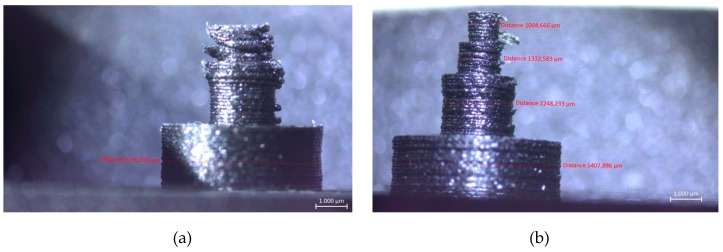
A detailed view of the benchmark part showing the bosses positioned on top of each other with nominal dimensions of (from bottom to up) 5 mm, 2 mm, 1 mm and 0.5 mm (**a**) Left figure shows the resulting geometry when “expand thin features” is not turned on (**b**) Right figure shows the resulting geometry when “expand thin features” option is activated.

**Figure 7:**
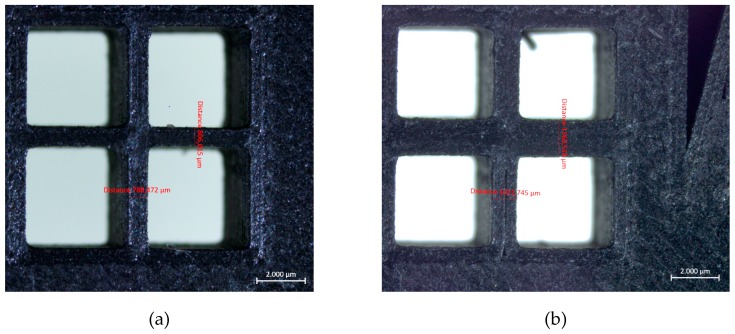
A detailed view of the benchmark part showing thin walls having a thickness of 1 mm (**a**) Left figure shows the resulting geometry when “expand thin features” is not turned on (**b**) Right figure shows the resulting geometry when “expand thin features” option is activated.

**Figure 8:**
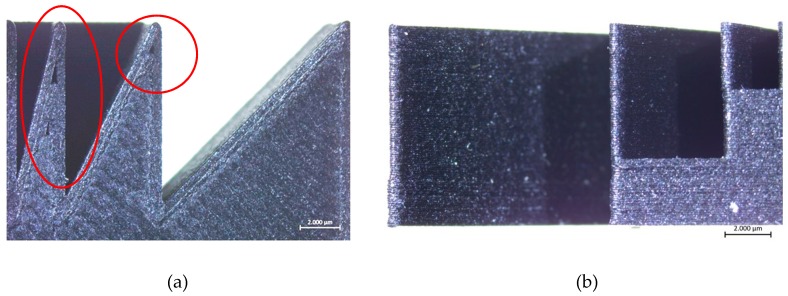
Sharp corners on the part (**a**) top view; (**b**) right side view.

**Figure 9:**
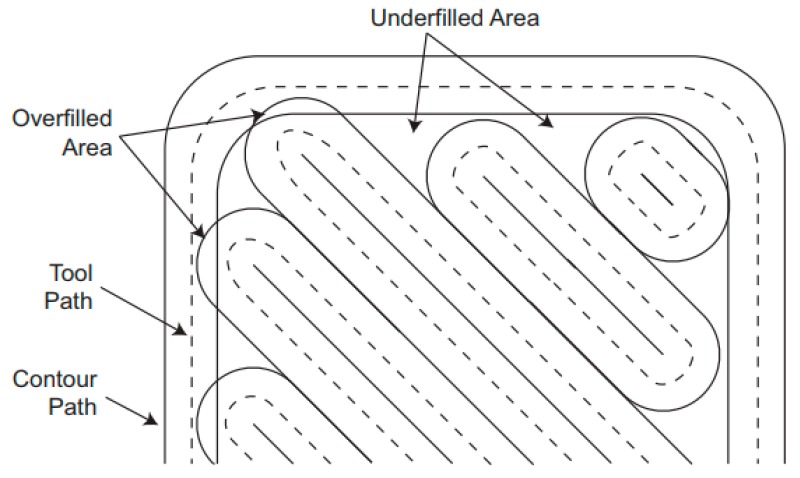
Illustration of the path influence when seeking to obtain geometric precision or mechanical performance [32].

**Figure 10:**
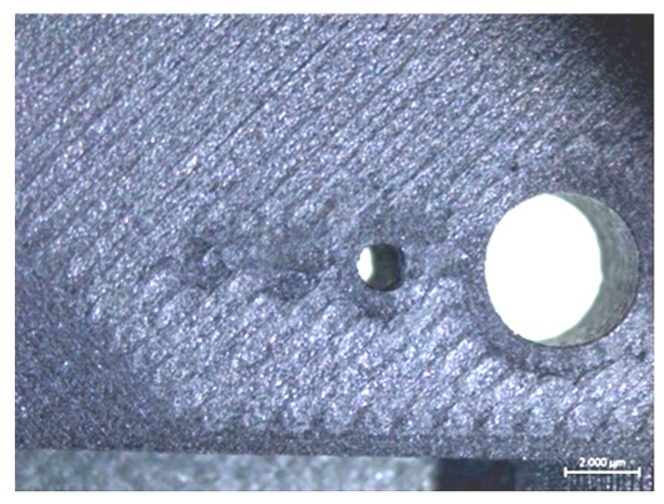
Circular vertical holes left and overhang surfaces right.

As mentioned before, the Benchmark-1 geometry was created to assess different AM technologies for metallic materials in terms of small bosses/holes, thin walls, stepping effect, surface quality, sharp corners, etc. [30]. Although there were some studies focusing on the dimensional accuracy of FFF parts [31], no study was found regarding carbon-fiber-reinforced nylon material. In the pre-processing software for the utilized equipment, the option of “expand thin features” was enabled. Without this option, thin walls with a thickness of 250 µm could not be built. This option also had an effect on the small bosses positioned on top of each other (see Figure 6). The outer cylinder had a nominal diameter of 5 mm, whereas the diameters of upper cylinders decreased from 5 mm to 2 mm, 1 mm and 0.5 mm. Figure 6a clearly shows that the bosses with a diameter of less than 2 mm could not be realized without activating the option of “expand thin features”. When this option was active (see Figure 6b), the obtained diameters, were measured as approximately 5.41 mm, 2.25 mm, 1.33 mm to 1 mm. Thin bosses could be made at a cost of losing dimensional accuracy for larger bosses since the diameter of 5 mm boss was measured to be 5.13 mm leading to a difference of 280 µm of difference with respect to the diameter measured with “expand thin features”. This observation was also valid with thin walls. A thin wall having a nominal thickness of 1 mm was measured to have a thickness 1.56 mm in one direction and 1.022 mm in the perpendicular one provided that “expand thin features” was turned on. However, without this option, thin walls of a thickness of 1 mm were measured to be 0.806 and 0.788 mm in two directions as depicted in Figure 7. These results led to the result that “expand thin features” option had a significant effect on features with dimensions less than 2 mm and could significantly alter the obtained dimensional accuracy as well as its change along various directions. In Figure 8, as another detailed view of the benchmark part, sharp corners with the “expand thin features” option can be observed from top and side views. The first important phenomenon to be observed was the lack of fusion toward the end of the sharp corners giving some porous areas, which would behave as weak points in the part performance. The reason for such an occurrence was due to the deposition strategy, which is illustrated in Figure 9 [32,33]. However, the sharpness that was produced was quite good from both views; it is even comparable to features obtained with metallic AM systems. As the last feature from the benchmark part, the circular holes with diameters of 5, 2, 1, and 0.5 mm were intended to be fabricated as shown in Figure 10 (left). However, only the holes with diameters of 5 and 2 mm could be produced with success. Smaller holes were totally blocked. 

**Figure 11:**
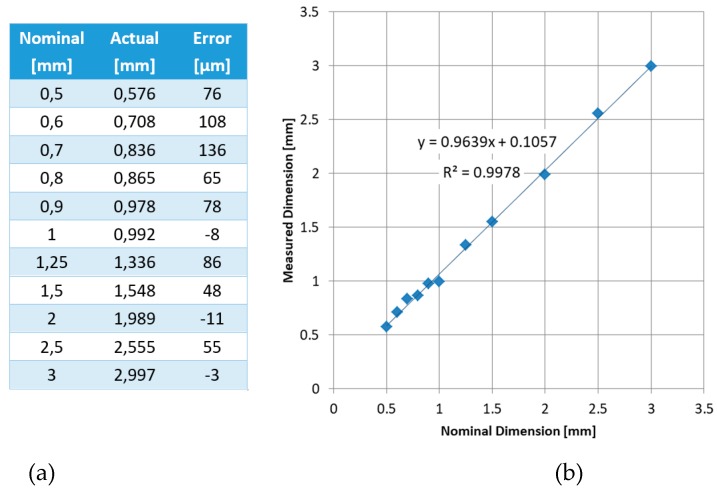
Nominal versus actual wall thickness values measured on a stereomicroscope (**a**); graphical demonstration of the results (**b**).

**Figure 12:**
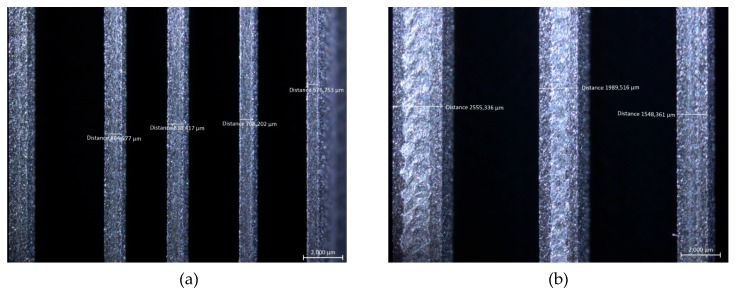
Thin wall features in Benchmark-2, nominal thickness values of (**a**) 0.5, 0.6, 0.7 and 0.8 mm; (**b**) 1.5, 2 and 2.5 mm.

The capability of fabricating thin walls with varying thickness values was demonstrated on the second benchmark as well as the effect of the inclination angle on the stair-stepping effect. Walls with thicknesses of 0.3–3.0 mm were planned with a height and width of 12 and 100 mm, respectively. Unfortunately, the walls with thicknesses of 0.3 and 0.4 mm were impossible to be made. As Figure 11 shows, the deviation of the wall thickness from the nominal value had a decreasing trend as the thickness increased. It should be noted that the deviations were generally on the positive side, meaning the walls were almost always thicker than intended (see Figure 12). The trend line fitted to the data had a very high R^2^ value of 0.9978, leading to the fact that the error can be compensated by making the wall thicknesses lower than desired, such that the final wall thickness matched the desired value with a lower error. 

**Figure 13:**
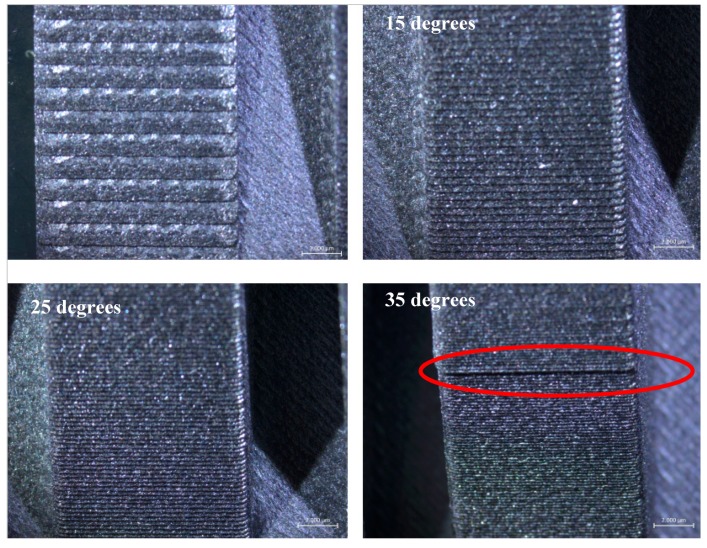
Stair effect in Benchmark-2 under different angles of inclination.

Figure 13 depicts the stair effect evident mostly as the angle of inclination gets decreased. The stair effect, inherent to layered manufacturing, deteriorates the surface quality and dimensional accuracy. As mentioned in Reference [34], the stair effect is mainly a function of the layer thickness and the angle of inclination. If the layer thickness is increased or the inclination angle is reduced, the stair effect becomes more dominant. This was clearly observed in Benchmark-2 as shown in Figure 13. Moreover, in the wall with an inclination of 35 degrees, a bump was observed as shown by an ellipse in the figure. The height of this bump’s location corresponded to the height of 12 mm, which was equal to the height of the walls manufactured together with these inclined blocks. The reason for such a geometrical inaccuracy may be attributed to the significant change in thermal input and resulting shrinkage; however, this needs to be investigated further.

### 3.2. Tensile and Hardness Testing

**Figure 14:**
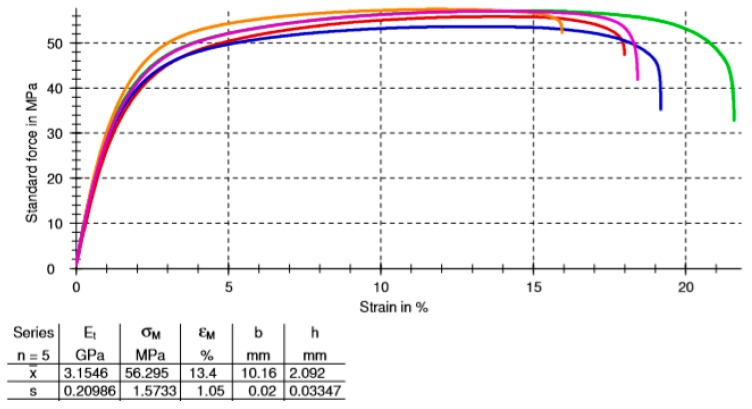
The tensile results of five repetitions of B_Onyx_R_100_XZ specimens; the table shows the averages and standard deviations of tensile properties.

**Figure 15:**
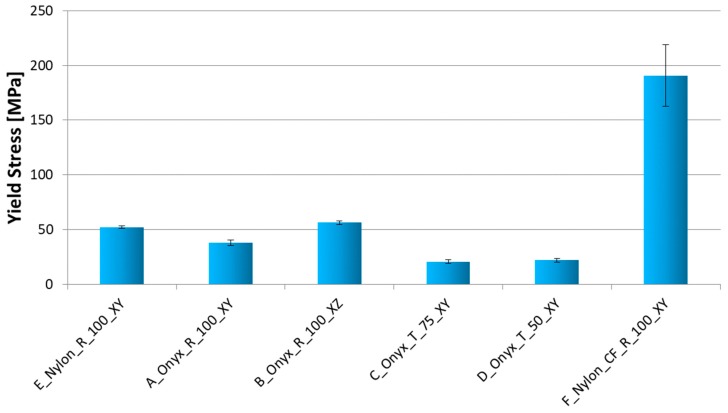
Tensile Test Results–Yield Stress.

**Figure 16:**
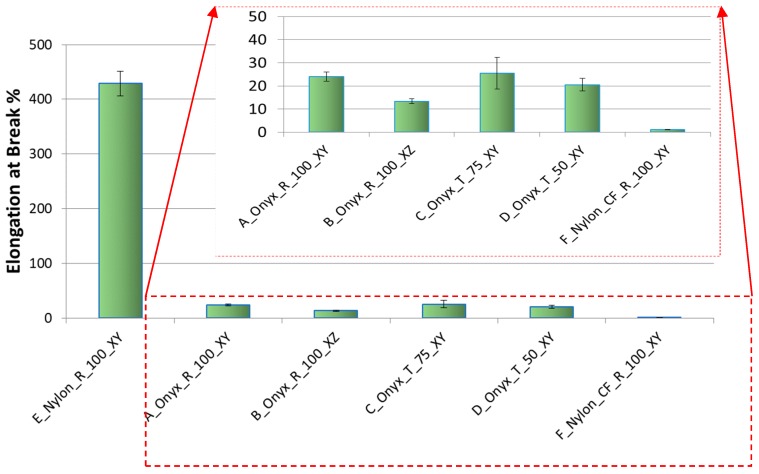
Tensile Test Results–Elongation at break.

**Figure 17:**
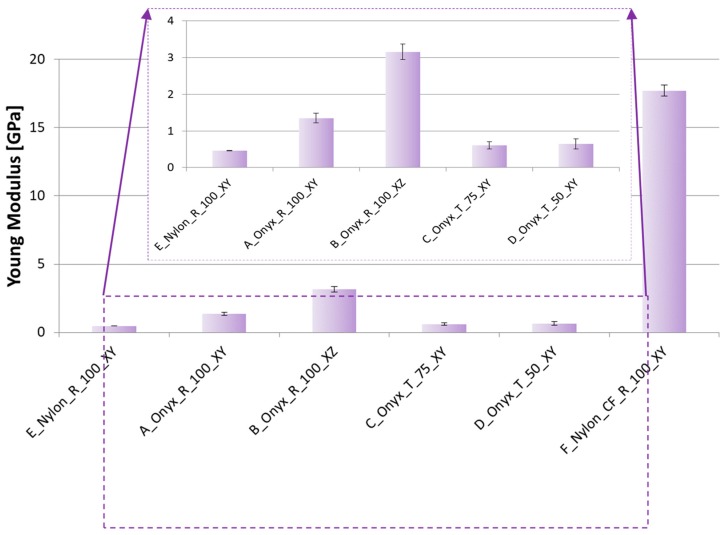
Tensile Test Results–Young’s Modulus.

**Figure 18:**
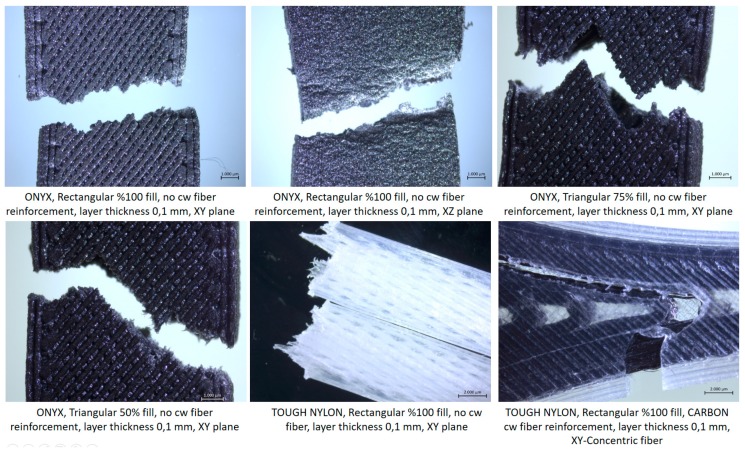
Fractured specimens after tensile testing.

The strain-stress curves for a specific configuration (B_Onyx_R_100_XZ specimen) are given in Figure 14. The repeatability of the tensile testing was satisfactory. The comparison of obtained results with respect to each other is given in Figure 15, Figure 16 and Figure 17 for yield stress, elongation at break and modulus of elasticity, respectively. As evident from these figures, continuous carbon reinforced nylon specimens yielded a severely higher modulus of elasticity and yield stress at a cost of almost no elongation at break. With continuous fiber-reinforcement, almost four-fold higher yield stress (190 MPa) was achieved in comparison to the highest yield stress achieved with chopped carbon fiber-reinforced specimens (56 MPa). A similar increase rate was achieved in the modulus of elasticity (from 3.15 GPa to 17.7 GPa). However, the elongation at break deteriorated from 25% to 1% when continuous chopped fibers were reinforced rather than chopped fibers. Moreover, it should be noted that all specimens broke at their minimum cross-sections while the continuous fiber-reinforced specimens broke at a higher position due to the weak interface between the fiber and matrix, as shown in Figure 18.

Compared to XY specimens, XZ specimens showed an increased Young’s modulus and yield stress, again at a cost of reduced elongation at break, as expected. Compared to nylon specimens with no reinforcement, chopped fiber-reinforced specimens built in the same direction (lying specimens on the XY plane) showed an almost three-fold higher elasticity modulus (1.35 GPa versus 0.47 GPa). On the other hand, the yield stress of chopped fiber-reinforced specimens was about 25% less than that of nylon (38 MPa versus 51 MPa). The advantage of using nylon in comparison to chopped carbon-reinforced nylon lies in the elongation at break, which exhibited a significant difference of 429% versus 24%, as shown in Figure 16.

The effect of build orientation could be seen by comparing the results of A_Onyx_R_100_XY and B_Onyx_R_100_XZ specimens. Lying specimens (XY) showed 30% less yield stress compared to standing specimens (XZ). This effect was more pronounced for Young’s Modulus. XY specimens showed a modulus of elasticity of 1.35 ± 0.13 GPa, whereas XZ specimens exhibited a modulus of elasticity of 3.15 ± 0.20 GPa. On the other hand, XY specimens broke at almost two-fold higher elongation values compared to XZ specimens. The other build direction where the tensile direction coincided with the build direction could not be tested due to the specimen’s dimensions exceeding the build direction. Yet, a recent study by Yasa showed that the impact toughness of chopped fiber-reinforced nylon produced in a similar manner to this study was severely affected when the impact was taken in between built layers leading to significant anisotropy in the obtained toughness [12].

Regarding the Young’s Modulus and yield stress, the density effect from 75% to 50% was about 5–6%. Yet, the density changing from 100% to 75% resulted in a higher variation, close to 25% in elongation at break. For porous materials, it is expected that the Young’s modulus and strength increase as the density increases since the amount of material available to bear the load is higher [35,36,37]. However, due to the specimen’s geometry being very thin, limiting the effect of the infill density, the porosity effect was almost negligible in terms of Young’s modulus and strength. Yet, the elongation at break changed from 25% to 20% when the infill density changed from 75% to 50%. The hardness of the chopped fiber-reinforced nylon was measured to be 71 Shore D. 

### 3.3. Microscopy

**Figure 19:**
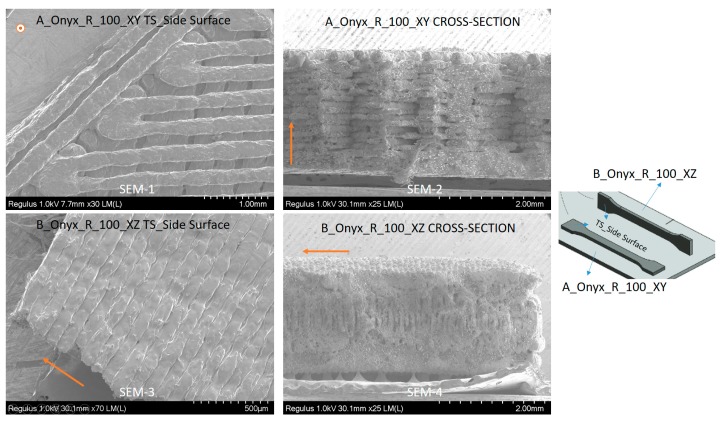
SEM graphs of A_Onyx_R_100_XY and B_Onyx_R_100_XZ specimens, orange arrows show the build direction.

**Figure 20:**
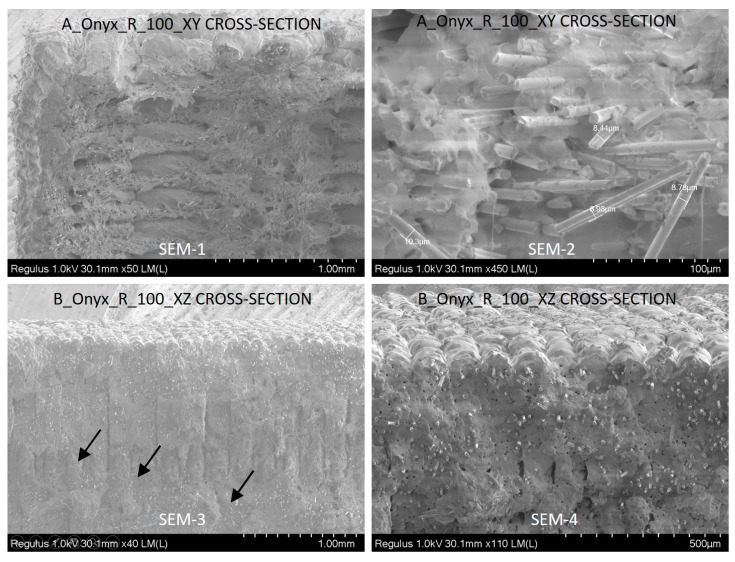
SEM graphs of A_Onyx_R_100_XY and B_Onyx_R_100_XZ specimen fracture surfaces with different magnifications.

The samples produced along different directions (A_Onyx_R_100_XY and B_Onyx_R_100_XZ) were observed with a scanning electron microscope to observe the differences. As seen in Figure 19 (SEM-1), the top side of the tensile specimen showed the top surface of the flat specimens where the deposited tracks of the material could be distinguished easily. A major amount of inter-layer porosity in between successive tracks was obvious. The cross-section of flat specimens (XY specimens) showed a fracture surface (SEM-2) where successive layers of deposited material were clear. In every layer, the deposition angle was switched from 45 to 135 degrees, making infill vectors in consecutive layers orthogonal to each other. When the fracture surface was magnified, the broken carbon fibers could easily be noticed (see Figure 20, SEM-1). Chopped carbon fibers having an approximate diameter of 9 µm were in line with the axis of infill vector. This mean value fell in the advertised data (10 ± 2 µm) [38]. Moreover, the individual carbon fibers apparent in Figure 18 SEM-2 may be proof of poor wetting by the matrix material.

For the specimens entitled B_Onyx_R_100_XZ, which were built on their thin edges, as shown in Figure 17 (SEM-3), the cross-section included a higher number of layers, as can be observed. It was not even possible to see the deposited tracks in the cross-section (SEM-4), as was the case with flat specimens (A_Onyx_R_100_XY SEM-2). However, the layers were visible in both views. The side surface of the tensile specimen (SEM-3) more obviously exhibited the layers with an approximate layer thickness of about 100 µm, which was well in line with the set value.

**Figure 21:**
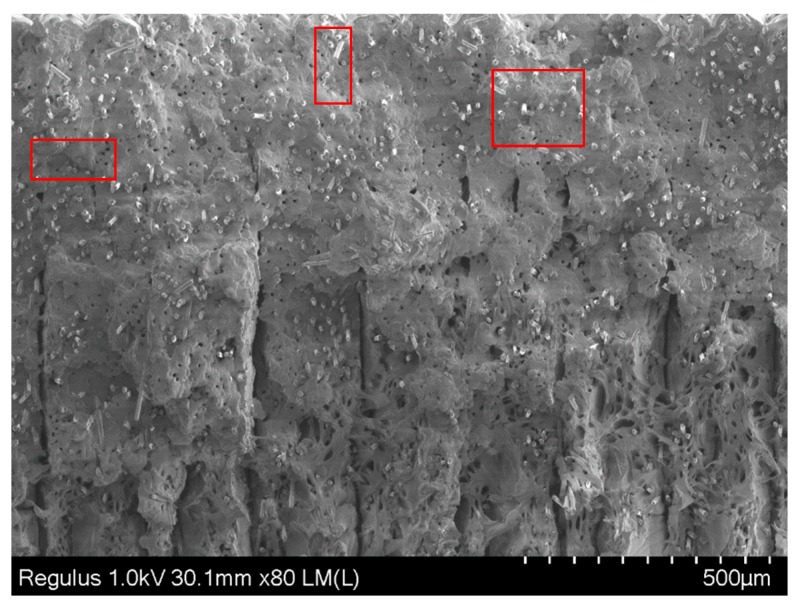
SEM graph of B_Onyx_R_100_XZ specimen fracture surface with 80× magnification; red rectangles show the voids due to fiber pull out.

The magnified graphs of the side surfaces and cross-sections of the tensile specimens given in Figure 18 yielded other observations. The crack-like porosities in between successive layers were visible in both specimens, as shown with black arrows (Figure 18 SEM-3). The contours scanned around the part to be filled in every layer limited these porosities. Due to the fact that the specimens were quite thin, the effect of dense contours probably contribute to a higher Young’s modulus and yield strength (see Figure 15 and Figure 17). The rectangles in Figure 21 indicate the voids due to fiber pull out. Moreover, fiber failure/fracture and small voids were observed as failure modes. As shown in Figure 16, the elongation at break was higher for flat specimens (A_Onyx_R_100_XY) than specimens built on their thin edges (B_Onyx_R_100_XZ). This was also evident from the SEM graphs.

## 4. Conclusions

This paper presented a study on the dimensional accuracy and mechanical properties of carbon fiber-reinforced nylon matrix composites produced by fused filament fabrication for use in real-life applications. The benchmark geometries produced from the chopped fiber-reinforced nylon matrix composites showed that the process is quite stable, and that the dimensional accuracy can be enhanced for some features using appropriate compensation techniques. The minimum feature size was observed as 2 mm for holes and bosses. The tensile test results showed that, compared to nylon without any reinforcement, the yield strength and modulus of elasticity were greatly enhanced at the cost of ductility. The enhancement was further increased when the carbon fibers were placed in a continuous manner. However, these samples showed almost no elongation at break. Scanning electron microscopy images of the fracture surfaces of tensile specimens indicated poor wetting of fibers based on the matrix material, fiber pullout, and spherical voids, as well as porosities in between deposited tracks as a manufacturing defect. Due to the build volume limitations, the testing could not be carried out on specimens where the axis of tension is parallel to the build axis. Actually, these are considered as the specimens where the maximum difference in mechanical properties is expected to be observed. Future work will focus on testing sub-size specimens and correlating the microstructural observations to the manufacturing defects as well as repeatability of tests. 

## Figures and Tables

**Table 1:** Tensile Specimen Fabrication Parameters.

No.	Part Name	Material	Infill	% Density	Fiber	Layer Thickness	Build Direction
1	E_Nylon_R_100_XY	NYLON	Rectangular	100%	None	0.1 mm	XY plane
2	A_Onyx_R_100_XY	ONYX	Rectangular	100%	None	0.1 mm	XY plane
3	B_Onyx_R_100_XZ	ONYX	Rectangular	100%	None	0.1 mm	XZ plane
4	C_Onyx_T_75_XY	ONYX	Triangular	75%	None	0.1 mm	XY plane
5	D_Onyx_T_50_XY	ONYX	Triangular	50%	None	0.1 mm	XY plane
6	F_Nylon_CF_R_100_XY	NYLON	Rectangular	100%	Carbon	0.125 mm	XY plane
